# Validation of travel times to hospital estimated by GIS

**DOI:** 10.1186/1476-072X-5-40

**Published:** 2006-09-19

**Authors:** Robin Haynes, Andrew P Jones, Violet Sauerzapf, Hongxin Zhao

**Affiliations:** 1 School of Environmental Sciences, University of East Anglia, Norwich NR4 7TJ, UK; 2 Respiratory Diseases Department, Centre for Infections, Health Protection Agency, 61 Colindale Avenue, London NW9 5EQ, UK

## Abstract

**Background:**

An increasing number of studies use GIS estimates of car travel times to health services, without presenting any evidence that the estimates are representative of real travel times. This investigation compared GIS estimates of travel times with the actual times reported by a sample of 475 cancer patients who had travelled by car to attend clinics at eight hospitals in the North of England.

**Methods:**

Car travel times were estimated by GIS using the shortest road route between home address and hospital and average speed assumptions. These estimates were compared with reported journey times and straight line distances using graphical, correlation and regression techniques.

**Results:**

There was a moderately strong association between reported times and estimated travel times (r = 0.856). Reported travel times were similarly related to straight line distances. Altogether, 50% of travel time estimates were within five minutes of the time reported by respondents, 77% were within ten minutes and 90% were within fifteen minutes. The distribution of over- and under-estimates was symmetrical, but estimated times tended to be longer than reported times with increasing distance from hospital. Almost all respondents rounded their travel time to the nearest five or ten minutes. The reason for many cases of reported journey times exceeding the estimated times was confirmed by respondents' comments as traffic congestion.

**Conclusion:**

GIS estimates of car travel times were moderately close approximations to reported times. GIS travel time estimates may be superior to reported travel times for modelling purposes because reported times contain errors and can reflect unusual circumstances. Comparison with reported times did not suggest that estimated times were a more sensitive measure than straight line distance.

## Background

Geographical access to health services is now often measured using car travel time estimates calculated in a geographical information system [1]. In such studies, the GIS is generally used to find the shortest travel time from each population location to each health facility along the road network. This process utilises information about road length and average travel speeds along successive segments of road. Travel time estimates have not replaced the use of straight line distances [2], but road distance and time are recognized as more appropriate measures of the travel effort actually experienced than straight line distances, particularly in regions with a patchy road network, physical barriers such as major rivers or hills or an irregular coastline [3].

The use of car travel time estimates has become commonplace in studies of variations in access to health care services [4-9] and in studies which evaluate the effect of geographical accessibility on the use of services [10-12] or on health outcomes [13]. They are also employed in studies which predict the impact of service reconfiguration on patient flows [14,15].

In spite of the widespread acceptance of GIS estimates of car travel times as a suitable measure of geographical access, there have been few attempts to establish the validity of the method. Evidence that variations in the use of health services are more strongly associated with estimated travel times than with straight line distance [10] seems to justify the increasing tendency for researchers to estimate travel times as the best single measure of the actual costs and inconvenience that people experience. But are GIS estimates of travel time accurate? Little attention has been given to that question.

One exception was an early study demonstrating the contribution that a GIS approach could make to assessing the cost of travel to people who visited recreation sites by car [16]. This study compared GIS-estimated travel times with the travel times actually reported by visitors to a recreation site and found very similar mean times, with closely comparable statistical distributions. The authors concluded that the GIS-based method provided a more accurate basis for cost estimates than the reported travel times in the majority of cases because of the very strong tendency for respondents to round travel times up or down. The exceptions were six families out of a sample of 351 parties, who reported much longer travel times than those estimated by the GIS method. They were believed to be "meanderers" who had not chosen to drive by the most direct route. The study's aim was to demonstrate that conventional pre-GIS estimates of travel effort made using straight line distances from the centroids of cities or counties produced overestimates of true times, and that this bias could be removed by calculating travel distances or times from residential locations correct to 1 km along the road network in a GIS. Mean travel times estimated by GIS were shown to be not significantly different from those reported by respondents, but the detailed distribution of differences between estimated travel times and perceived times was not discussed.

In cases where the decision to attend hospital for treatment is made by the patient, it is possible that utilisation is more strongly influenced by accessibility as perceived by the patient than accessibility modelled by GIS. In a recent study [17] residents of Caerphilly county borough, Wales, were asked how well placed they considered their home to be for the nearest hospital with a casualty department. Perceived ease of access, measured on a five point Likert scale from "not at all well placed" to "very well placed", was positively related to travel time estimates made in a GIS (Spearman's rank correlation r = 0.38). Perceived access was similarly related to the straight line distance to hospital, so the researchers argued that there may be little advantage in using the sophisticated GIS measure in preference to the more-easily-calculated straight line distance in studies that aim to capture the perception of local residents. They conceded that it cannot be assumed that either perceived accessibility or GIS assessments of travel time are good predictors of the actual travel experiences of patients.

Most large population studies of health service accessibility rely on estimated travel times as a substitute for direct observations of actual travel effort, which would be difficult to obtain. Hitherto, researchers have assumed that travel time calculations based on road networks and average road speeds have provided unbiased estimates of real travel times, but there is little published evidence to support the assumption. In this study, we aim to contribute some evidence, by comparing GIS estimates of car travel times with subjects' recall of actual journey times. Our sample was of people with cancer who attended clinics at various hospitals in the North of England. The objective was to investigate whether car travel times estimated in a GIS are good approximations to the times that people report about an actual journey and whether they may be used with confidence to represent unmeasured real car travel times. We were also interested in assessing whether travel time estimates were more successful in capturing the variations between real journey times than straight line distances, which are much easier to calculate.

## Methods

Patients undergoing treatment or management for breast, colorectal, lung, ovary and prostate cancer and attending a hospital clinic for a routine post-diagnosis appointment were asked to complete a short questionnaire form about their travel to the hospital on that occasion. This study was part of a larger investigation of the influence of geographical access on survival from cancer, and these cancer types were chosen because they are sites where early intervention is known to affect outcome, or are sites where earlier studies have suggested access may be significant. Patients resident in the study area (Northern England, defined by the boundaries of the Northern and Yorkshire Cancer Registry) were asked to state their home postcode and identify their mode of transport to hospital, whether they had travelled there directly from home and give the time taken for the journey (and parking time separately if they had travelled by car). They were also invited to comment on any difficulties of the journey. Eight different hospitals were selected to give a variety of large specialized cancer centres and more local clinics in different parts of Northern England. The sample was not intended to be representative of all cancer patients, but to cover the spectrum of travel experiences from journeys across extensive, sparsely-populated rural tracts, through small towns and within dense urban conurbations, as far as possible. Questionnaires were handed to patients waiting for their appointments by receptionists or research nurses at each suitable clinic.

The postcode of residence of each respondent was converted to a grid reference using a published look-up table [18] and hospital locations were similarly converted into grid references. Estimates of car travel times from patients' homes to the hospital they attended were made using the Ordnance Survey Meridian 1:50,000 scale digital road network. Average car speeds for different road classes in urban and rural settings (Table 1) were used to calculate how long it would take to drive along any particular road segment. These average speeds were the same as those used by previous workers [19], who derived them from UK Department of Transport figures [20] adjusted to fit a range of test car journeys. For every patient-to-hospital journey, the shortest travel time estimate was recorded. Straight line distances to hospital were also calculated from the grid coordinates of patients' residences and hospitals.

**Table 1:** Road speed estimates used to calculate car travel times

**Road Type**	**Average Road Speed (mph)**	
	
	**Rural**	**Urban**
Motorway	63	35
A-Road Primary Dual Carriageway	54	28
A-Road Dual Carriageway	50	25
A-Road Primary Single Carriageway	45	25
A-Road Single Carriageway	32	18
B-Road Dual Carriageway	36	18
B-Road Single Carriageway	24	12
Minor Road	14	11

The GIS estimates of car travel times from home to hospital were then compared with the reported journey times and the straight line distances, for the same patients, using correlation and regression techniques. The distribution of differences between reported and estimated times was examined, and possible reasons for the differences were investigated. The comparisons were restricted to every respondent who travelled by car to hospital (their own car, being given a lift or by taxi) who said they travelled straight to the hospital from home.

## Results

Overall, out of 780 questionnaire forms handed out, 696 were completed, a response rate of 89%. Each hospital produced between 69 and 116 completed questionnaire forms, so the respondents were drawn from a variety of destinations with none dominating the sample. Respondents were more likely to be female (70%) than male (30%), reflecting the preponderance of female cancer sites in the sample of clinics. The age range extended from 18 years to 75 years and over, but the most common age category was 45–64 years (47%).

Travelling in the household car (65% of patients) or being given a lift by someone known to the patient (17%) were the most frequently used modes of transport to the hospital. Altogether 87% of the sample travelled by car (including taxis and hospital cars). Travel by bus was the second most popular category, but bus transport was used by only 5% of the sample. Very small proportions of patients used other modes. Most patients (95%) had gone straight to the hospital from their homes. Out of 696 respondents, 475 had travelled by car directly from home to hospital and had supplied information about their travel time.

Distances and travel times to hospital were all positively skewed, with a majority of patients having short journeys, but a smaller number needing to travel much further. For the respondents who had travelled straight to hospital by car, the mean straight line distance between home and hospital was 15.0 km, ranging from 400 m to 106 km (Table 2). The mean reported and estimated journey times were almost identical (28.1 and 28.2 minutes respectively) and the other descriptive measures were comparable for reported and estimated times. The reported travel times did not include the time it took to park the car at the hospital. The mean reported parking time was 7.4 minutes, but was substantially more for some patients. Altogether, 34% said it had taken ten minutes or more to find a parking space at the hospital and 4% claimed parking had taken them at least 30 minutes. Inclusion of the parking time with the reported travel time would have distorted the comparison with the GIS estimates, which did not include parking time.

**Table 2:** Straight line distances, reported travel times and estimated travel times to hospital: descriptive statistics.

	Mean	Standard deviation	Minimum	Quartile 1	Median	Quartile 2	Maximum
Straight line distance (km)	15.0	14.2	0.4	5.4	11.2	20.8	106
Reported travel time (mins)	28.1	18.4	4.0	15.0	20.0	35.0	120
Estimated travel time (mins	28.2	19.0	1.8	15.0	23.9	37.9	129

**Figure 1:**
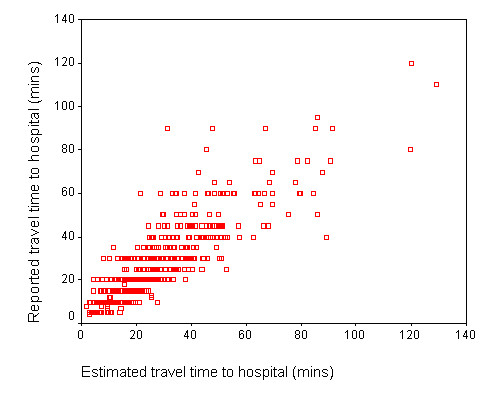
Comparison of estimated and reported journey to hospital times for patients who travelled by car.

For the patients who travelled directly to hospital by car, Figure 1 shows that there was a fairly close correspondence between estimated and reported car journey time to hospital. The correlation between reported travel time and estimated travel time was 0.856 (p < 0.001). The strongest association was between straight line distance to hospital and estimated travel time (r = 0.935, p < 0.001). The association between straight line distance and reported travel time (0.858, p < 0.001) was almost identical in strength with the association between estimated and reported travel time.

Fitting a linear regression line to Figure 1 gave an intercept constant of 5.119 (standard error 0.778) and a gradient of 0.825 (standard error 0.023). The positive intercept and slope less than one indicated a tendency for journeys estimated at close to 0 minutes to be reported as 5 minutes and for reported times to be less than directly proportional to the GIS estimates.

**Figure 2:**
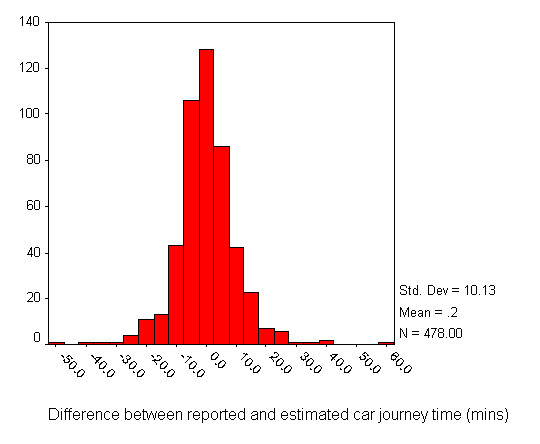
Distribution of the differences between reported and estimated car journey time (minutes).

The differences between reported journey times and their corresponding estimated times were identified by subtracting the estimated time from each reported time. Figure 2 shows that most of the differences between reported journey times and these adjusted estimated times were close to 0 minutes (the mean was 0.2 minutes), and that there was an approximately symmetrical distribution, indicating that overestimates and underestimates were made with almost equal likelihood. Altogether, 50% of travel time estimates were within five minutes of the time reported by respondents, 77% were within ten minutes and 90% were within fifteen minutes. There were a few extreme differences of up to one hour.

**Figure 3:**
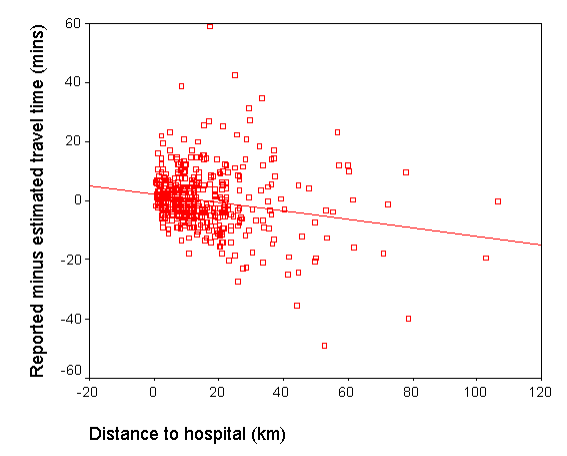
Differences between reported and estimated travel times as a function of distance to hospital.

Figure 3 shows the differences between reported and estimated journey times as a function of straight line distance to hospital. There was a slight negative correlation (r = -0.205, p < 0.001). The trend line had an intercept of 2.313 (standard error 0.655) and a gradient of -0.144 (standard error 0.032), indicating a tendency for the difference between values to diminish by 0.14 minutes every kilometre. It is evident from Figure 3 that the slope of the trend line was influenced by a few outliers at long distances from hospital.

Only one respondent said that their journey had taken less than 5 minutes, while 22 patients estimated exactly 5 minutes. As can be seen in the clustering of travel times in horizontal lines in Figure 1, one reason for a lack of correspondence between reported and estimated travel times was that almost all respondents reported a journey time either rounded to a ten minute interval (61% of responses) or to an intervening five minute interval (37% of responses).

**Figure 4:**
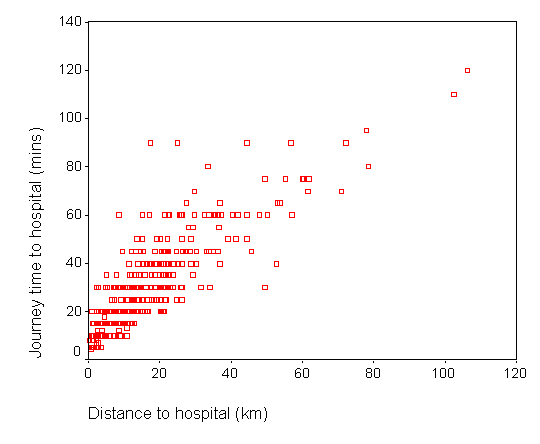
Reported car travel times to hospital and straight line distance.

A probably much larger contributing factor was the variability of reported travel times for similar journey distances. Figure 4 shows the relationship between reported travel times and distance for direct car journeys. For any given straight line distance, a range of actual travel times was reported. Even at very low distances of a few kilometres, respondents reported car journey times between 5 and 30 minutes.

Many journeys with the same straight line distance in Figure 4 represent routes that included very different road types. To minimize this source of variation, an attempt was made to identify groups of respondents who lived close together and therefore might be expected to have used much the same route when travelling to hospital. Clusters of respondents were first identified by mapping the grid coordinates of their residential postcodes and selecting those living within one kilometre of others. Only respondents who travelled directly to the same hospital by private car were included. Table 3 summarises the journey characteristics for 12 such clusters, representing various distances from hospital.

**Table 3:** Range of travel times for similar journeys

Journey	Straight distance range (km)	Estimated time range (mins)	Reported time range (mins)
1 (N = 3)	2.0 – 2.2	4 – 7	5 – 10
2 (N = 3)	2.1 – 2.7	11 – 14	5 – 20
3 (N = 4)	6.8 – 7.1	15 – 16	10 – 15
4 (N = 4)	6.9 – 7.6	21 – 24	20 – 30
5 (N = 6)	9.1 – 9.9	20 – 24	15 – 30
6 (N = 3)	12.0 – 12.2	22 – 23	15 – 30
7 (N = 6)	12.0 – 12.8	18 – 21	15 – 25
8 (N = 4)	12.9 – 13.6	20 – 21	15 – 25
9 (N = 5)	14.7 – 15.3	32 – 35	25 – 60
10 (N = 6)	21.0 – 21.4	38 – 40	20 – 45
11 (N = 4)	21.9 – 22.5	38 – 39	35 – 50
12 (N = 2)	28.7 – 29.5	29 – 30	50 – 60

With proximate starting points and less than one kilometre variation in straight line distances to hospital, Table 3 demonstrates that variations of up to three minutes in the estimated journey time to hospital were common, irrespective of journey length. Reported journey time variations were always greater, and much greater in some cases. The most extreme example was five patients living in close proximity (group 9) whose journey times were estimated as between 32 and 35 minutes, but who reported times from 25 minutes to one hour. It is clear that journeys probably made over the same roads could produce very different actual travel experiences.

Examination of the comments made on the questionnaire forms by respondents revealed some of the individual circumstances that might have been responsible for a mismatch in particular cases. The worst anomaly was for a female patient aged 45–64 years attending hospital in her own household car whose estimated journey time was 30 minutes, but who reported a time of 90 minutes. This patient wrote "Traffic holdups on the road" when asked to comment on any difficulties with the journey. The second highest positive difference was a female aged 65–74 years, whose estimated car travel time was 47 minutes, but who also reported a 90 minute journey. This patient said her journey had been "very difficult" and, when asked to explain any problems, said "Getting into the hospital". It was not clear whether this referred to travel in the city in the vicinity of the hospital or to problems with parking. The same patient said it had taken 25 minutes to park, so perhaps she had mistakenly included this time in her journey time of 90 minutes. One other patient who had taken 90 minutes to arrive (with an estimated time of 67 minutes) commented "Traffic congestion...", with the name of a small town where the problem was encountered. Another, whose journey was estimated as 42 minutes, took 70 minutes (with an additional 20 minutes to park) and commented "Morning traffic, queue for parking". Respondents at the negative extreme of Figure 2 with reported journey times that were much shorter than estimated made no comments to explain why they had relatively speedy journeys, so we were not able to explore the reasons. Many of these patients had been estimated to take an hour or more, but reported journeys of 40–60 minutes with short parking times, perhaps indicating unusually good traffic conditions and higher than average driving speeds.

## Discussion

Amongst our sample of patients, GIS estimates based on average car speeds on different classes of road and cancer patients' reports of the time taken to make actual car journeys to hospital were closely related. There was no evidence, however, that GIS estimates were better than straight line distance in representing the variations in journey times reported by patients.

This study had a number of methodological limitations. The sample of cancer patients was not representative of hospital patients in general because only 30% were men. This is not likely to be a serious source of bias because the men and women in the sample had very similar mean driving distances, reported times and estimated times. We also examined the differences between reported and estimated travel times for men and women separately and found no significant difference between the means. The high response rate makes it seem unlikely that the sample was biased in other ways (for example in socio-economic terms), although we were unable to check this.

The method of calculating travel times in a GIS is subject to a number of errors and uncertainties. Although we had access to the widely used Ordnance Survey Meridian digital road network, the product is digitised at a scale of 1:50,000, so the representation of roads is simplified, generally shortening them slightly compared to their true lengths. Furthermore we were not able to take into account the presence of topographic features like hills that may influence vehicle travel speeds. We did not have information on the actual location of patient residences, but that of the first delivery address in their postcode. In rural areas a single postcode can cover a large area, and this can lead to inaccuracies in the identification of patient outset locations [21].

We are not confident that the recall of car travel time by respondents was accurate. One large source of variation was the almost universal practice (followed by 98% of respondents) of rounding journey times to the nearest five or ten minutes. In addition, the survey design might have contributed to other kinds of error. The questionnaire forms were completed in hospital waiting rooms by patients who were about to consult a doctor about a serious illness. These stressful conditions might have caused confusion or inaccuracies of recall. Although respondents were requested not to include the time taken to park at the hospital in their journey time, it is possible that some patients did so. The difficulty in finding a parking space at the hospital was the most frequent negative comment made by respondents, and several reported long periods of time waiting in a queue or driving around searching for a space. Perhaps these experiences affected the memory of the journey as a whole for some respondents.

Travel times and the relationship between travel times and straight line distance are undoubtedly context-dependent. Our study area combined a mixture of dense urban areas, lowland farming districts and sparsely-settled hills, crossed by rivers and lakes and bounded by a coastline. In other settings, particularly those dominated by difficult mountain terrain or a highly crenulated coast, the relationships might be expected to be different (see, for example [22]). The resources available did not permit us to compare a more detailed breakdown of journeys such as urban versus rural routes, but this might be a worthwhile avenue for future investigation.

Altogether 87% of the sample travelled to hospital by car (including taxis and hospital cars), suggesting that estimated car travel times to hospital are likely to be suitable measures of journey time for most cancer patients attending a consultation (and perhaps out-patients with other conditions attending hospital) in the UK. Travel by bus was the second most popular category, but bus transport was used by only 5% of the sample. Very small proportions of patients used other modes. Attempts to include public transport in GIS measures of accessibility to health services involve time-consuming procedures [3,4] and, in some circumstances, might not be worth the effort.

It is clear that journeys over the same roads could produce very different actual travel experiences, probably depending on the characteristics of individual drivers, their vehicles, the volume of traffic at the time, the occurrence of road works and other driving conditions including the weather. Travel times estimated electronically in a GIS can do no more than aim to capture the average situation. For most applications, however, the average situation is an appropriate measure. Large studies of the equity of service provision across a region, the effects of accessibility on the utilization of services and the impact of poor service access on health do not generally have direct information about actual travel times to draw on, and must rely on estimates. A single value is usually required to represent travel impedance between each origin and destination. The average journey time is generally more useful for modelling purposes than the range of possible extremes. If the range of possible travel experiences is of interest, then the results of this study give an idea of what might be expected. Of course, even under average conditions, peoples' reported travel times contain rounding errors and possibly some recall error. It cannot be assumed that reported times are necessarily more accurate than computer estimates.

We found a moderately strong and statistically significant correspondence between the actual car travel times to hospital reported by patients and estimated travel times calculated in a GIS from patients' postcodes and information about the road types connecting them to the hospital. Unusual traffic delays accounted for the most extreme discrepancies. Because actual journey times were subject to many more volatile influences than estimates based on average driving speeds, it is not surprising that the association between them was less than perfect. Overall, journeys where the reported time exceeded the estimated time were balanced by similar numbers of journeys with the difference reversed, but there was a slight tendency for the reported time to be longer close to the hospital and shorter at greater distances. This appeared to be at least partly due to the common practice of rounding short car journeys up to five minutes and the presence of a few extreme values of reportedly fast journeys to distant hospitals.

It was surprising that GIS estimates based on average car speeds on various road types were no better than straight line distances in predicting reported journey times in the North of England, although others have reached a similar conclusion in a different region [17]. Since all three measures involved errors, and the errors were different, it is not possible to conclude that one or other was the better measure for the study area. Straight line distance is easily calculated and does not need a GIS platform, so is preferable in that respect. In some other geographical contexts, of course, the advantages of a GIS measure might be more decisive.

## Conclusion

These results contribute evidence to support the widespread use of GIS estimates of travel time in health geography studies. A comparison between GIS estimates based on average car speeds on different classes of road and cancer patients' reports of the time taken to make actual car journeys to hospital has shown that the two were closely related. The computer estimates were within ten minutes of reported times for 77% of car journeys to hospital, but the differences between estimated and reported times were weakly associated with distance to hospital. Reported times contained rounding errors and probably some recall errors, and were much affected by traffic conditions on the day. The GIS estimates of travel times were not necessarily less accurate than the reported times, and they were more appropriate for modelling purposes because they represented average conditions. We found no evidence, however, that GIS estimates were better than straight line distance in representing the variations in journey times reported by patients.

## Competing interests

The author(s) declare that they have no competing interests.

## Authors' contributions

RH drafted the manuscript and contributed to analysis and interpretation. AJ directed the project and contributed to analysis and interpretation. VS performed the GIS operations and contributed to analysis and interpretation. HZ constructed the GIS database, organized and managed the patient survey and coded the responses. All agreed the final manuscript.
